# Supplementary material for: Development and Characterization of a Molecularly Imprinted Polymer for the Selective Removal of Brilliant Green Textile Dye from River and Textile Industry Effluents
Source: Polymers (Basel). 2023 Sep 8;15(18):3709. doi: 10.3390/polym15183709 (PMC10535355; doi:10.3390/polym15183709)
Supplement: Supplementary file 1 [file polymers-15-03709-s001.zip › polymers-2551394-supplementary.pdf]

Supplementary Material

Development and Characterization of a Molecularly Imprinted Polymer for the Selective Removal of Brilliant Green Textile Dye from River and Textile Industry Effluents

Miguel Luna Quinto [1], Sabir Khan [1,2,3,4], Jaime Vega-Chacón [1], Bianca Mortari [2], Ademar Wong [2,3], Maria Del Pilar Taboada Sotomayor [2,3] and Gino Picasso [1],*

[1] Technology of Materials for Environmental Remediation Group (TecMARA), Faculty of Sciences, National University of Engineering, Lima 15333, Peru

[2] Institute of Chemistry, State University of São Paulo (UNESP), Araraquara 14800-900, SP, Brazil

[3] National Institute of Alternative Technologies for Detection, Toxicological Evaluation and Removal of Micropollutants and Radioactives (INCT-DATREM), Araraquara 14800-900, SP, Brazil

[4] Department of Natural Sciences, Mathematics, and Statistics, Federal Rural University of the Semi-Arid, Mossoro 59625-900, Brazil

* Correspondence: gpicasso@uni.edu.pe

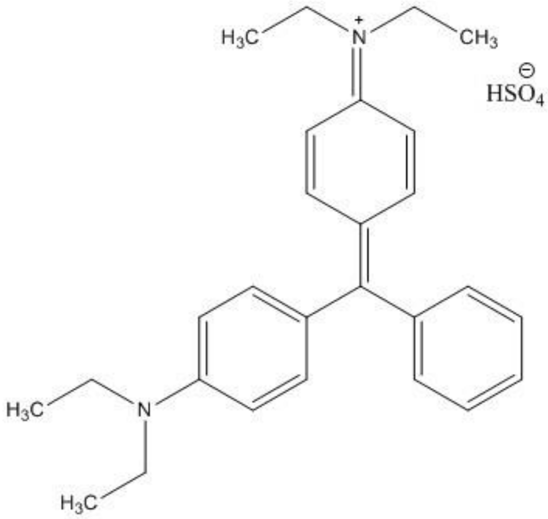

Figure S1. Chemical structure of Brilliant Green.

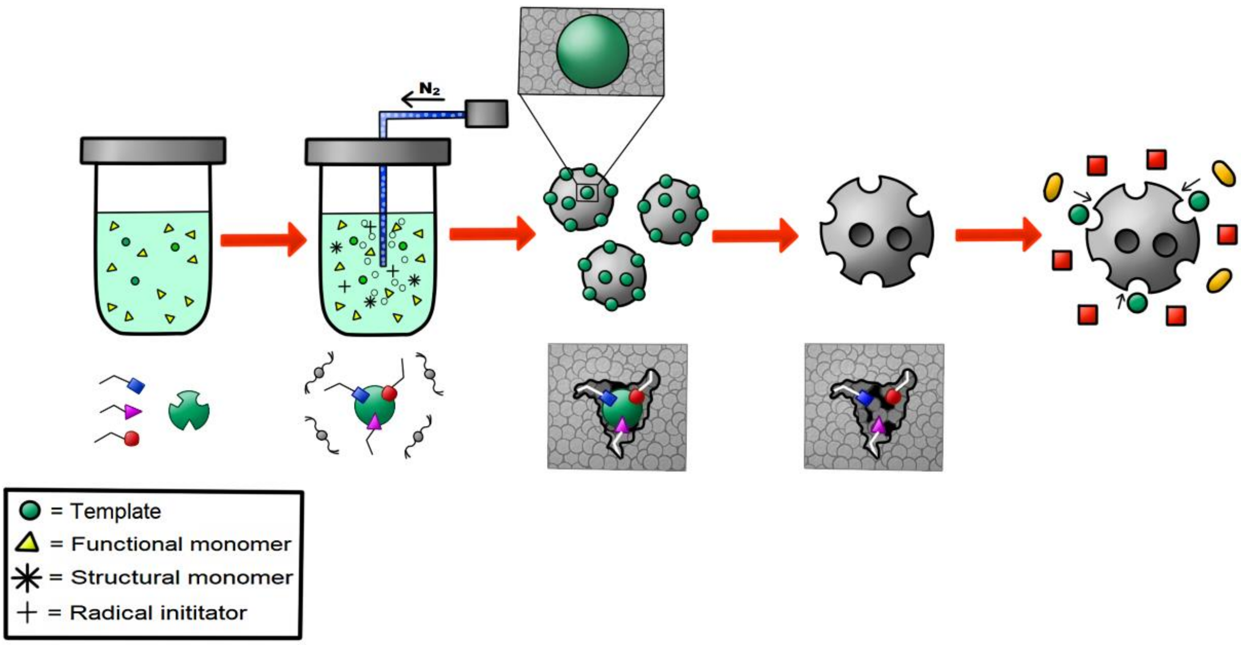

Figure S2. Schematic illustration of MIPs preparation.

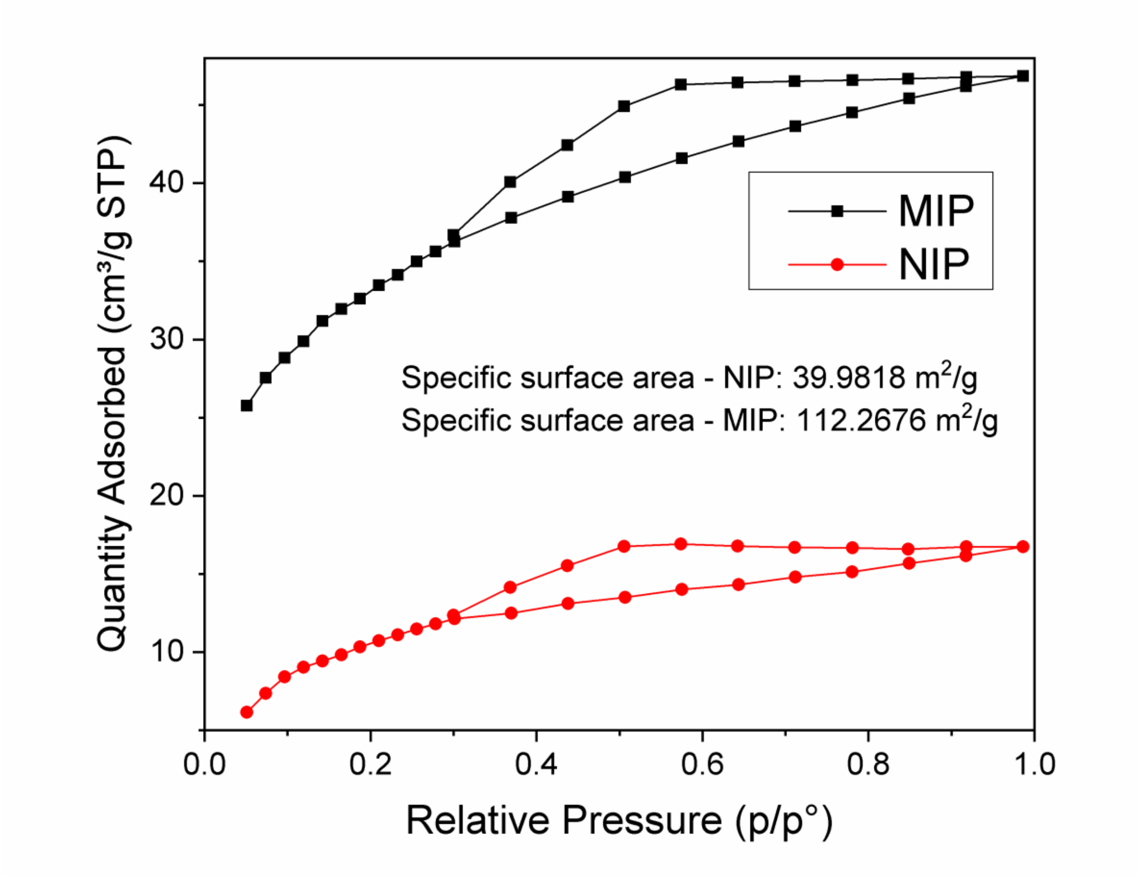

Figure S3. Nitrogen adsorption-desorption isotherms obtained for the MIP and NIP investigated.

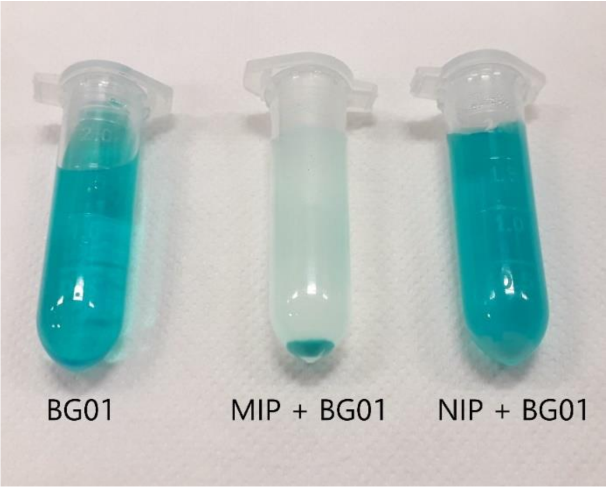

Figure S4. Photographs of the vials before and after adsorption of BG (at the concentration of 48.26 mg L^{-1}), along with the MIP and NIP.

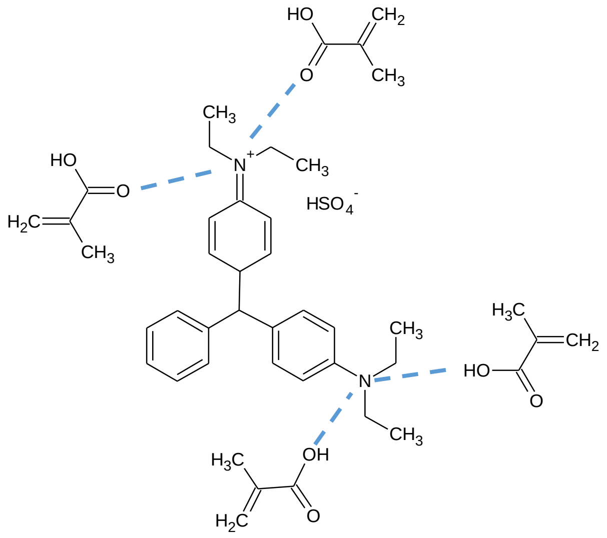

Figure S5. Diagram of the possible interactions considered in the computational simulation during the formation of the MIP selective toward BG.
